# Cerebral Air Embolism Triggered by Infection of a Pulmonary Cyst in the Context of Air Travel: A Case Report

**DOI:** 10.7759/cureus.89244

**Published:** 2025-08-02

**Authors:** Shinji Katayanagi

**Affiliations:** 1 Department of Respiratory Medicine, Kamagaya General Hospital, Chiba, JPN

**Keywords:** barotrauma, cerebral air embolism, pulmonary cyst infection, pulmonary venous-bronchial fistula, seizures

## Abstract

Cerebral air embolism (CAE) is a rare and potentially fatal event. While most cases result from iatrogenic causes, such as central venous catheterization, pulmonary sources, especially infected cysts, are scarcely reported. We describe a case of a previously healthy 61‑year‑old man who lost consciousness immediately after a flight. On admission, his Glasgow Coma Scale was E3V1M3, and CT and MRI revealed multiple cerebral air emboli. He was intubated and treated with mechanical ventilation, targeted temperature management, and levetiracetam. CSF analysis showed no pleocytosis, and EEG revealed no epileptiform discharges. Follow-up CT and MRI demonstrated decreased pneumocephalus but manifestation of ischemic foci. Although consciousness improved to E4V4M6 and extubation was achieved, left hemiparesis persisted. Imaging on admission identified a 7-cm fluid-filled emphysematous lung cyst adjacent to the inferior pulmonary vein, accompanied by elevated inflammatory markers, which normalized after antibiotics; however, the cyst remained unchanged. Echocardiography and whole-body CT excluded cardiac shunts or vascular malformations. Notably, the patient reported a similar episode of confusion following a flight more than 10 years earlier, during which imaging was unremarkable, suggesting that a combination of factors, including a lung cyst and infection, as well as cabin pressure changes, may have played the triggering role in CAE. This report highlights that infection of a pulmonary cyst can result in systemic air embolism, particularly under barometric pressure fluctuations. Patients with known pulmonary cysts, especially frequent flyers, should undergo proactive evaluation and management of structural lung lesions to prevent air embolism.

## Introduction

Cerebral air embolism (CAE) most commonly occurs due to iatrogenic interventions such as central venous catheterization or trauma; however, only a few cases have been linked to endogenous pulmonary source [1-3]. CAE is associated with considerable mortality and morbidity, with fatality rates as high as 40%-90%, and even among survivors, long-term neurological sequelae are frequent, including motor deficits, cognitive impairment, visual field loss, and seizures [2,4]. Pulmonary cysts, when infected or structurally compromised, can form bronchovenous fistulas, enabling air entry into systemic circulation [5,6]. Additionally, air travel introduces significant barometric pressure changes, which may precipitate CAE in susceptible individuals [7,8]. Here, we present a case of CAE attributed to an infected pulmonary cyst, triggered by air travel.

## Case presentation

**Figure 1:**
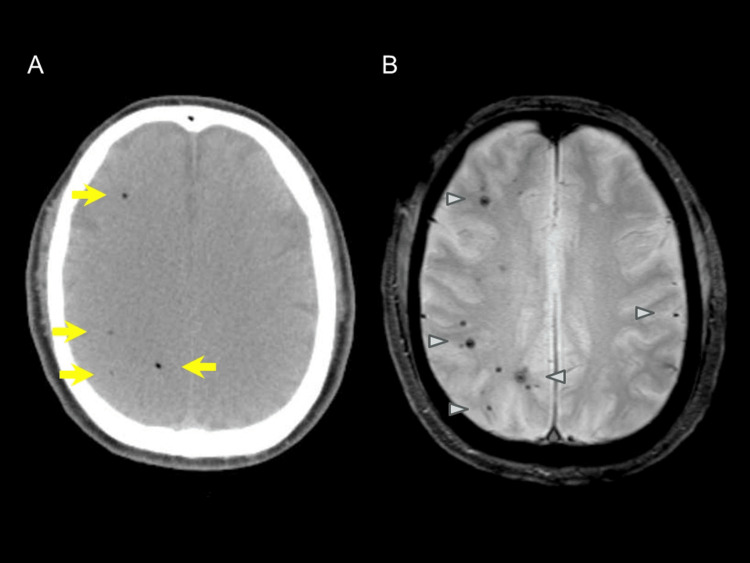
Brain CT and MRI on admission (Day 0). Brain CT on admission (Day 0) demonstrates multiple intravascular air foci within the right cerebral white matter (arrows), consistent with arterial cerebral air embolism (A). T2*-weighted MRI on Day 0 shows multiple hypointense spots (arrowheads) (B).

**Figure 2:**
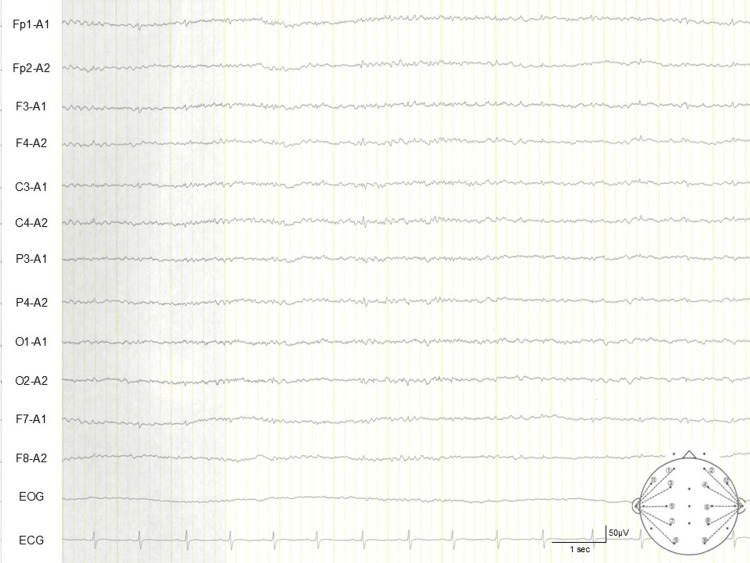
EEG and ECG on admission (Day 0). Representative EEG montage obtained at admission, demonstrating a reactive, normal synchronous background rhythm without epileptiform discharges or ictal patterns. Additionally, ECG shows a normal sinus rhythm without conduction abnormalities or arrhythmias. Fp: frontopolar; F: frontal; C: central; P: parietal; O: occipital; A: auricular (ear lobe electrodes); EOG: electrooculogram.

**Figure 3:**
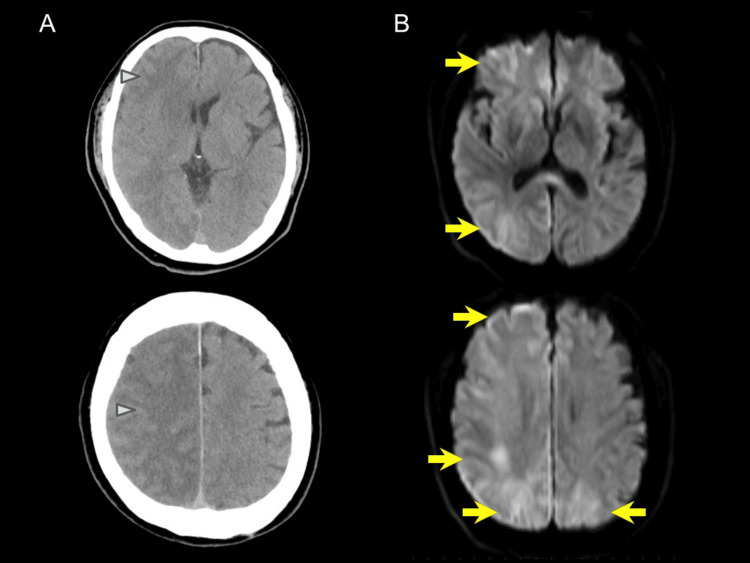
Follow-up brain CT and MRI. Brain CT on Day 6 shows ischemic lesions (arrowheads) and reduction in pneumocephalus (A). Diffusion-weighted MRI on Day 10 reveals multifocal high-intensity areas in the right cerebral hemisphere, left frontal lobe, and occipital lobe (arrows) (B).

A 61-year-old man presented with sudden impaired consciousness immediately after disembarking from a flight. On admission (Day 0), his Glasgow Coma Scale (GCS) was E3V1M3 [9], accompanied by right conjugate eye deviation and focal seizures in the left upper limb. Emergent brain CT revealed multifocal intracerebral air emboli, and T2*-weighted MRI on the same day demonstrated multiple hypointense foci, consistent with intraparenchymal air bubbles (Figures 1A, 1B). He was intubated and managed with mechanical ventilation, targeted temperature control, and levetiracetam (1 g/day). Cerebrospinal fluid was sterile without pleocytosis (Table 1), and EEG revealed no epileptiform discharges (Figure 2). Follow-up CT on Day 6 showed partial resolution of pneumocephalus (Figure 3A), while subsequent MRI on Day 10 detected multiple ischemic foci in the right cerebral hemisphere, left frontal lobe, and left occipital lobe (Figure 3B). His consciousness gradually improved, with a GCS score of E4V4M6, and extubation was performed on Day 10. However, left hemiparesis persisted, and manual muscle testing (MMT) of the left limb remained at 0-1, indicating severe neurological impairment. Consequently, prolonged rehabilitation was required, and he was transferred to another facility for continued rehabilitation on Day 63.

**Table 1:** Relevant laboratory findings along with their reference values.

Laboratory findings	Value	Reference value
Complete blood cell count		
White blood cell, 10^3^ cells/µL	12.78	3.3-8.6
Neutrophils, n (%)	82.5	38.5-80.5
Lymphocytes, n (%)	11.6	16.5-49.5
Monocytes, n (%)	4.1	2.0-10.0
Eosinophils, n (%)	1.3	0.0-8.5
Basophils, n (%)	0.5	0.0-2.5
Blood biochemistry		
C-reactive protein, mg/dL	9.42	0-0.14
Procalcitonin, ng/mL	0.24	< 0.05
Coagulation		
Fibrinogen, mg/dL	584.7	200-400
Cerebrospinal fluid		
Opening pressure, mmH₂O	235	60-250
Appearance	Clear	Clear
Density	1.006	1.005-1.007
White blood cell, cells/µL	2	< 5
pH	7.38	7.35-7.4
Lactate dehydrogenase, ng/mL	16.0	< 40
Total protein, mg/dL	33	15-45
Glucose, mg/dL	70	40-75
Bacterial culture	Negative	Negative

**Figure 4:**
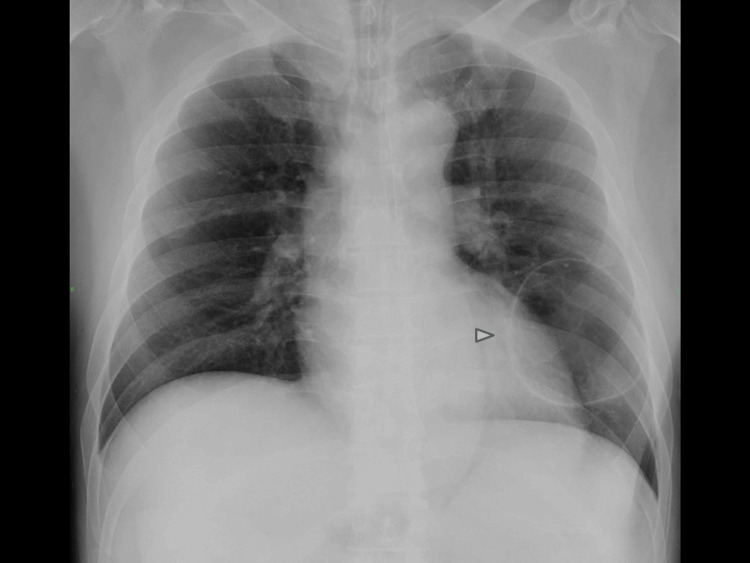
Chest radiograph on admission (Day 0). Post-intubation AP chest X-ray shows a large, well-circumscribed, thin-walled lucent cystic lesion in the left lower lung field (arrowhead). AP: anteroposterior projection.

**Figure 5:**
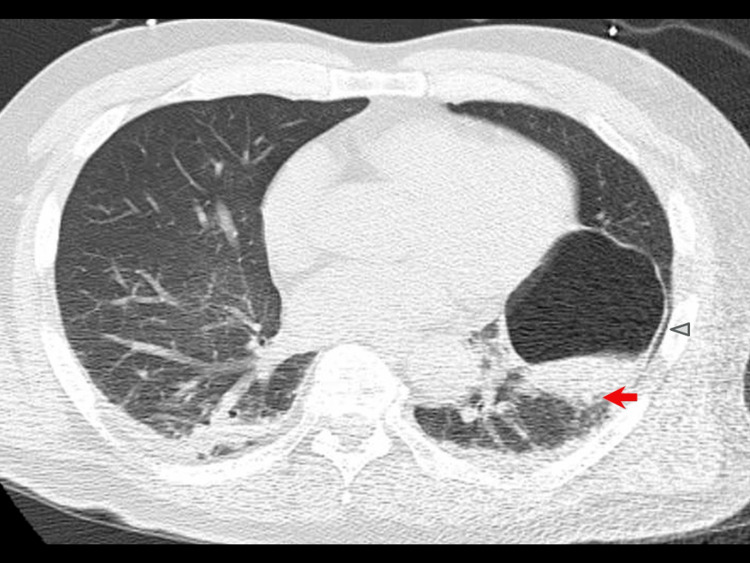
Chest CT on admission (Day 0). Chest CT on admission confirms a 7-cm thin-walled, fluid-filled emphysematous cyst located in the lingular segment of the left upper lobe (arrowhead). The dorsal wall of this cyst closely abuts the left inferior pulmonary vein (arrow).

On initial evaluation (Day 0), laboratory tests, chest X-ray, and chest CT demonstrated elevated inflammatory markers in sera and a 7-cm fluid-filled emphysematous lung cyst in the lingular segment adjacent to the left inferior pulmonary vein (Table 1; Figures 4, 5). Inflammatory markers and intracystic fluid were alleviated with sulbactam/ampicillin; however, structural cystic lesion remained visible on radiological imaging. Echocardiography and whole-body CT did not detect any cardiac shunts nor vascular malformations. Taken together, these findings suggest the presence of a bronchovenous fistula between the infected pulmonary cyst and pulmonary vein, providing a pathway for air entry leading to CAE. He was referred for surgical evaluation following stabilization.

**Figure 6:**
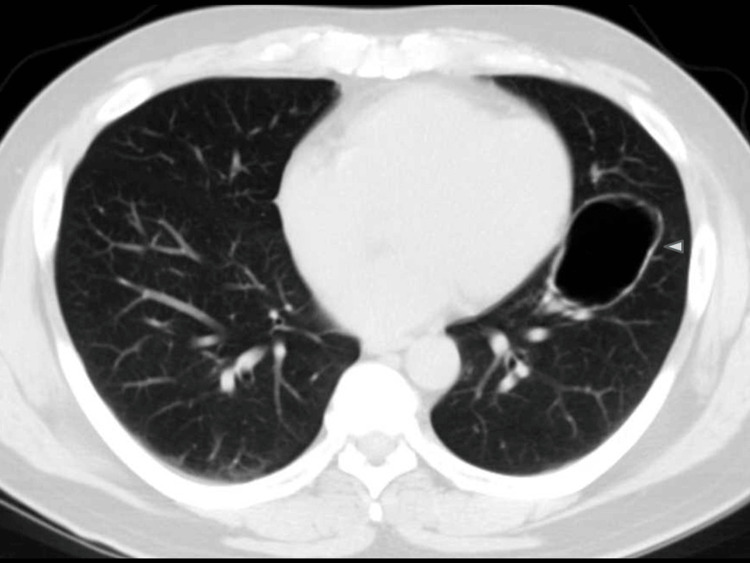
Historical chest CT. Chest CT performed over 10 years prior demonstrates a cystic lesion in the lingular segment of the left upper lobe (arrowhead), indicating a longstanding structural abnormality.

Notably, the patient had a history of frequent air travel for work and had experienced a similar episode of transient confusion following one air travel more than 10 years earlier. Brain imaging at that time showed no abnormalities; however, a lung cyst was pointed out (Figure 6). These findings suggest that in addition to the presence of lung cyst and pressure changes during the flight, a new trigger such as an infection, may have led to the development of CAE.

## Discussion

CAE primarily arises from iatrogenic causes, trauma, or pulmonary barotrauma [1-3]. However, endogenous pulmonary sources, including infected cysts, are scarcely reported [3]. This case highlights an uncommon mechanism: air entry into the systemic circulation via a bronchovenous fistula secondary to an infected lung cyst [6]. While CT pulmonary angiography could potentially visualize vascular communication, it was not pursued due to concerns regarding contrast use in the setting of acute neurological instability, use of mechanical ventilation, and the anticipated reduced diagnostic yield associated with the absence of barometric pressure changes after disembarking; instead, bronchovenous fistula was inferred from imaging anatomy and exclusion of other sources. Structurally, the proximity of the cyst to the pulmonary vein likely facilitated this connection, and infection or inflammation may have compromised the integrity of the cyst wall through enzymatic degradation or microerosion, enabling escape of air under pressure changes [7,8].

Barometric fluctuations during flight may have expanded the cyst, promoting air movement into the vasculature [10]. Similar pathophysiology has been described in cases of decompression-induced CAE due to pulmonary abnormalities [5,11]. Management of CAE includes high-flow oxygen, seizure control, and supportive care. Although hyperbaric oxygen therapy is the gold standard, it was not feasible in this unstable patient, consistent with prior reports in similar settings [2].

This case underscores the need for pre-travel evaluation for patients with known cystic lung disease. Given the potential for recurrence, prophylactic surgical resection may be considered in selected cases, especially individuals with repeated infection of known pulmonary cystic lesions or prior neurological symptoms [12,13]. While this represents a single case, further accumulation of such reports may inform clinical guidelines and preventive strategies.

## Conclusions

Infected pulmonary cysts may precipitate cerebral air embolism through bronchovenous fistulization, particularly under barometric stress such as during air travel. Clinicians should consider this rare but critical mechanism in patients with neurological symptoms following flights, and proactive management, including surgical intervention, should be considered for individuals with known pulmonary cysts and frequent flyers.

## References

[1] Muth CM, Shank ES (2000). Gas embolism. N Engl J Med.

[2] Červeňák V, Všianský V, Cviková M (2024). Cerebral air embolism: neurologic manifestations, prognosis, and outcome. Front Neurol.

[3] Togo M, Hoshi T, Matsuoka R, Imai Y, Kohara N (2017). Multiple small hemorrhagic infarcts in cerebral air embolism: a case report. BMC Res Notes.

[4] Chuang DY, Sundararajan S, Sundararajan VA, Feldman DI, Xiong W (2019). Accidental air embolism: an uncommon cause of iatrogenic stroke. Stroke.

[5] Desgranges FP, Cour M, Hernu R, Delafosse B, Argaud L (2013). Cerebral air embolism during an aircraft flight in a passenger with an air-filled lung cavity associated with remote lung surgery. J Thorac Cardiovasc Surg.

[6] Takizawa S, Tokuoka K, Ohnuki Y, Akiyama K, Kobayashi N, Shinohara Y (2000). Chronological changes in cerebral air embolism that occurred during continuous drainage of infected lung bullae. Cerebrovasc Dis.

[7] Arnaiz J, Marco de Lucas E, Piedra T, Arnaiz Garcia ME, Patel AD, Gutierrez A (2011). In-flight seizures and fatal air embolism: the importance of a chest radiograph. Arch Neurol.

[8] Farshchi Zarabi S, Parotto M, Katznelson R, Downar J (2017). Massive ischemic stroke due to pulmonary barotrauma and cerebral artery air embolism during commercial air travel. Am J Case Rep.

[9] Teasdale G, Jennett B (1974). Assessment of coma and impaired consciousness: a practical scale. Lancet.

[10] Lanfranco J, Romero Legro I, Freire AX, Nearing K, Ratnakant S (2017). Pulmonary air embolism: an infrequent complication in the radiology suite. Am J Case Rep.

[11] Uts A, Li D, Kurbanov D (2023). Retrograde cerebral air embolism associated with bronchovenous fistula. Cureus.

[12] Orford RR (1993). Preflight medical screening of patients. Chest.

[13] (2002). Managing passengers with respiratory disease planning air travel: British Thoracic Society recommendations. Thorax.

